# Supplementary material for: Selection for Translational Efficiency in Genes Associated with Alphaproteobacterial Gene Transfer Agents
Source: mSystems. 2022 Nov 14;7(6):e00892-22. doi: 10.1128/msystems.00892-22 (PMC9765227; doi:10.1128/msystems.00892-22)
Supplement: FIG S1 [file msystems.00892-22-s0001.pdf]

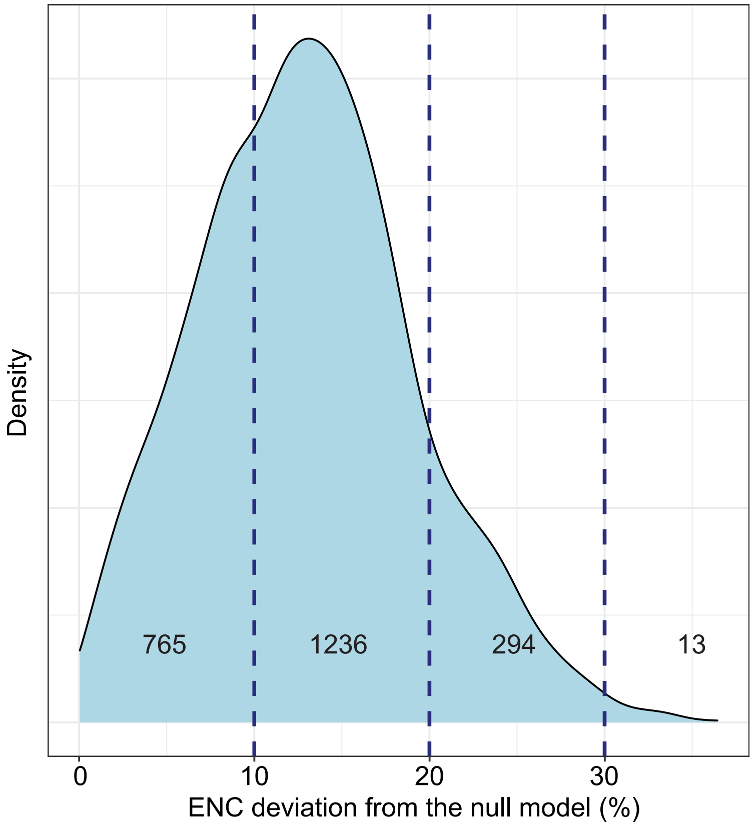

Supplemental Figure S1. Distribution of deviations of the effective number of codon (ENC) values from the expected ENC values under the null model of no codon bias. The distribution contains deviations for 2,308 reference GTA genes found in the 208 genomes. Numbers on the plot designate the number of reference GTA genes in an interval delineated by dashed lines.
