# Supplementary material for: Selection for Translational Efficiency in Genes Associated with Alphaproteobacterial Gene Transfer Agents
Source: mSystems. 2022 Nov 14;7(6):e00892-22. doi: 10.1128/msystems.00892-22 (PMC9765227; doi:10.1128/msystems.00892-22)
Supplement: FIG S2 [file msystems.00892-22-s0002.pdf]

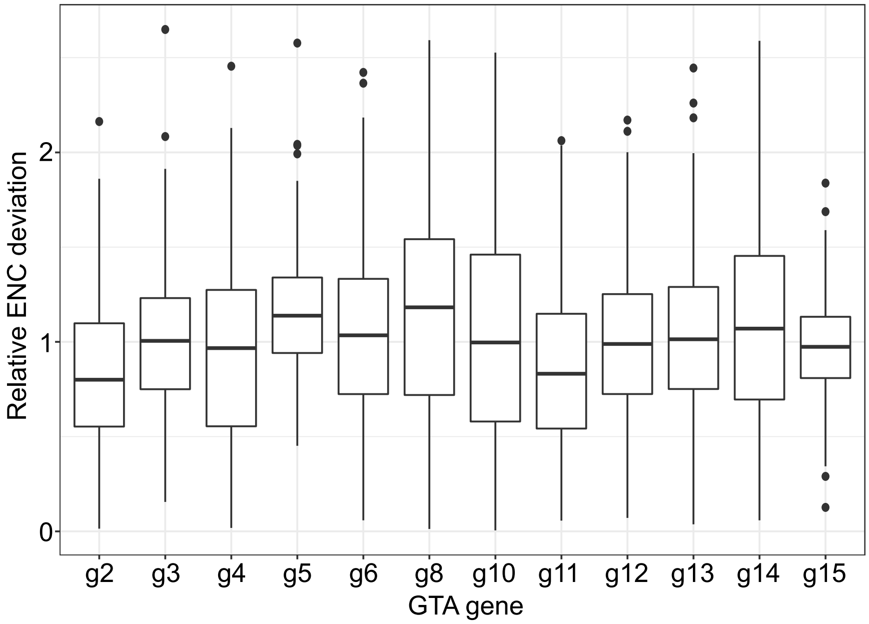

Supplemental Figure S2. Deviation of the effective number of codon (ENC) values for individual reference GTA genes in comparison to the genomic average. The deviation of the ENC from the expectation under the null model for each GTA gene was normalized by the average ENC deviation of its genome. Line within a box displays the median normalized ENC value for a GTA gene across all genomes. The boxes are bounded by first and third quartiles. Whiskers represent ptAI values within 1.5*interquartile range. Dots outside of whiskers are outliers.
