# Supplementary material for: Selection for Translational Efficiency in Genes Associated with Alphaproteobacterial Gene Transfer Agents
Source: mSystems. 2022 Nov 14;7(6):e00892-22. doi: 10.1128/msystems.00892-22 (PMC9765227; doi:10.1128/msystems.00892-22)
Supplement: FIG S3 [file msystems.00892-22-s0003.pdf]

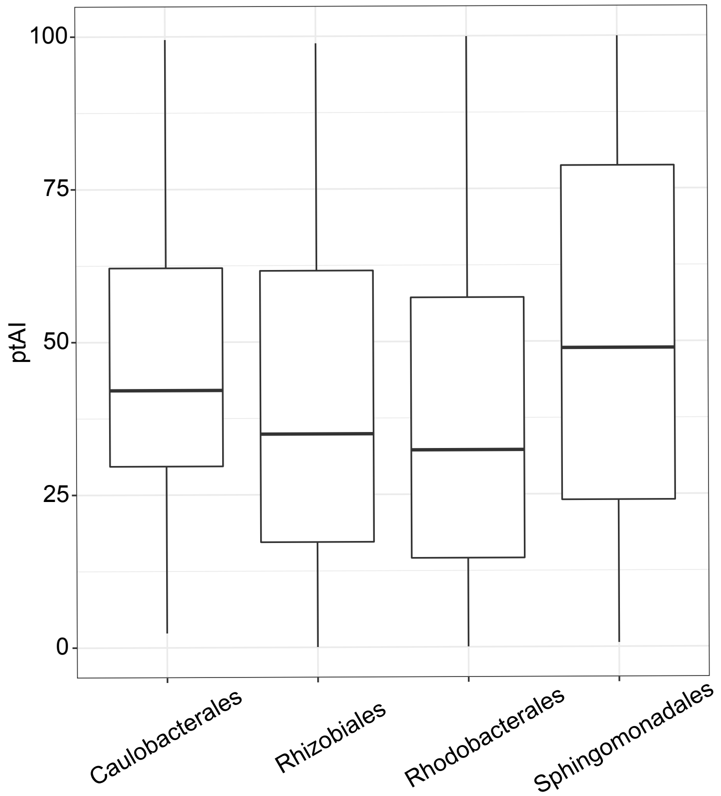

Supplemental Figure S3. Distributions of ptAI values in all reference GTA genes across four orders of the class *Alphaproteobacteria*. Line within a box displays the median ptAI value for a GTA gene across all genomes. The boxes are bounded by first and third quartiles. Whiskers represent ptAI values within 1.5*interquartile range. Dots outside of whiskers are outliers.
