# Supplementary material for: Selection for Translational Efficiency in Genes Associated with Alphaproteobacterial Gene Transfer Agents
Source: mSystems. 2022 Nov 14;7(6):e00892-22. doi: 10.1128/msystems.00892-22 (PMC9765227; doi:10.1128/msystems.00892-22)
Supplement: FIG S4 [file msystems.00892-22-s0004.pdf]

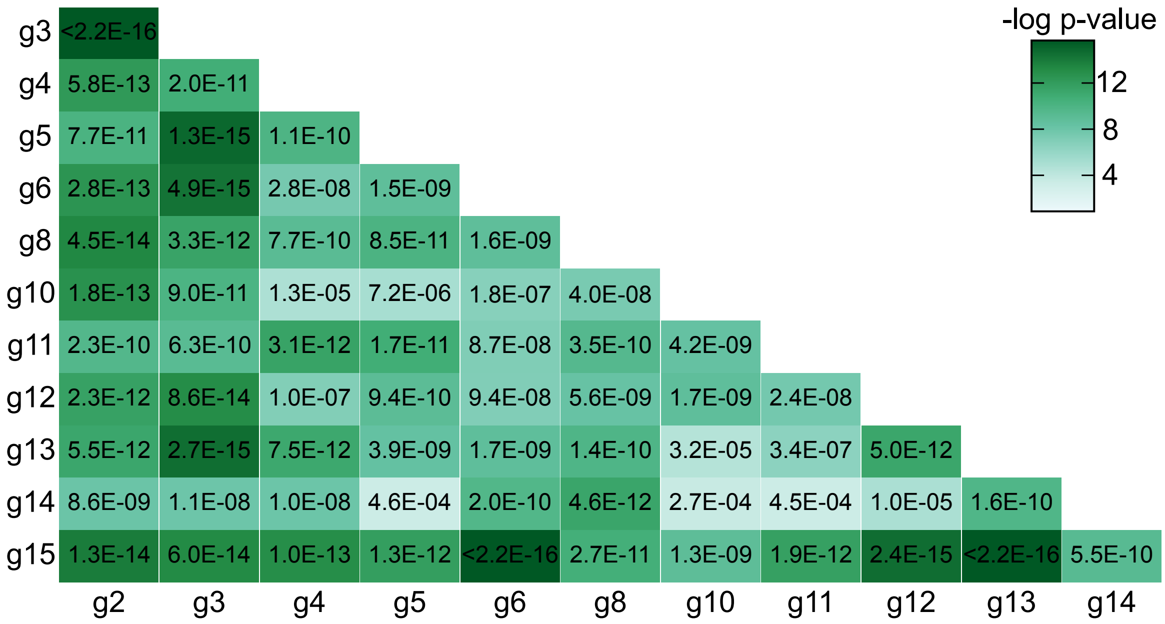

Supplemental Figure S4. PGLS model fit among ptAI values of the reference GTA gene pairs. Each pairwise comparison is represented by a rectangle that is color-coded according to the p-values from the PGLS analysis of the reference GTA gene pairs. The numerical p-values are listed within each rectangle.
