# Supplementary material for: Selection for Translational Efficiency in Genes Associated with Alphaproteobacterial Gene Transfer Agents
Source: mSystems. 2022 Nov 14;7(6):e00892-22. doi: 10.1128/msystems.00892-22 (PMC9765227; doi:10.1128/msystems.00892-22)
Supplement: TABLE S1 [file msystems.00892-22-s0007.pdf]

Supplemental Table S1. Four molecular pathways significantly over-represented in the protein-protein interaction network shown in Figure 3. The pathway information was obtained from KEGG database.

KEGG Pathway ID	Pathway name	p-value (after Benjamini-Hochberg correction)
sphm00900	Terpenoid backbone biosynthesis	0.0102
sphm03440	Homologous recombination	0.0119
sphm00906	Carotenoid biosynthesis	0.0202
sphm03430	Mismatch repair	0.0349
