# Supplementary material for: Selection for Translational Efficiency in Genes Associated with Alphaproteobacterial Gene Transfer Agents
Source: mSystems. 2022 Nov 14;7(6):e00892-22. doi: 10.1128/msystems.00892-22 (PMC9765227; doi:10.1128/msystems.00892-22)
Supplement: TABLE S2 [file msystems.00892-22-s0008.pdf]

Supplemental Table S2. Significance and slope of the fit of the phylogenetic generalized least squares (PGLS) models between the reference GTA genes and putative head-completion protein in *Sphingomonadales*. Statistically significant associations (p-value <0.05) are highlighted in orange.

Reference GTA gene	p-value	Slope
g2	0.82086	-0.03579
g3	0.59683	0.08254
g4	0.40305	0.13635
g5	0.21417	0.15219
g6	0.01450	0.41291
g8	0.83146	0.03253
g10	0.50256	0.17411
g11	0.00004	0.10553
g12	0.03593	0.25124
g13	0.85847	0.02868
g14	0.00170	0.59603
g15	0.00002	0.13779
