# Supplementary material for: Selection for Translational Efficiency in Genes Associated with Alphaproteobacterial Gene Transfer Agents
Source: mSystems. 2022 Nov 14;7(6):e00892-22. doi: 10.1128/msystems.00892-22 (PMC9765227; doi:10.1128/msystems.00892-22)
Supplement: FIG S5 [file msystems.00892-22-s0005.pdf]

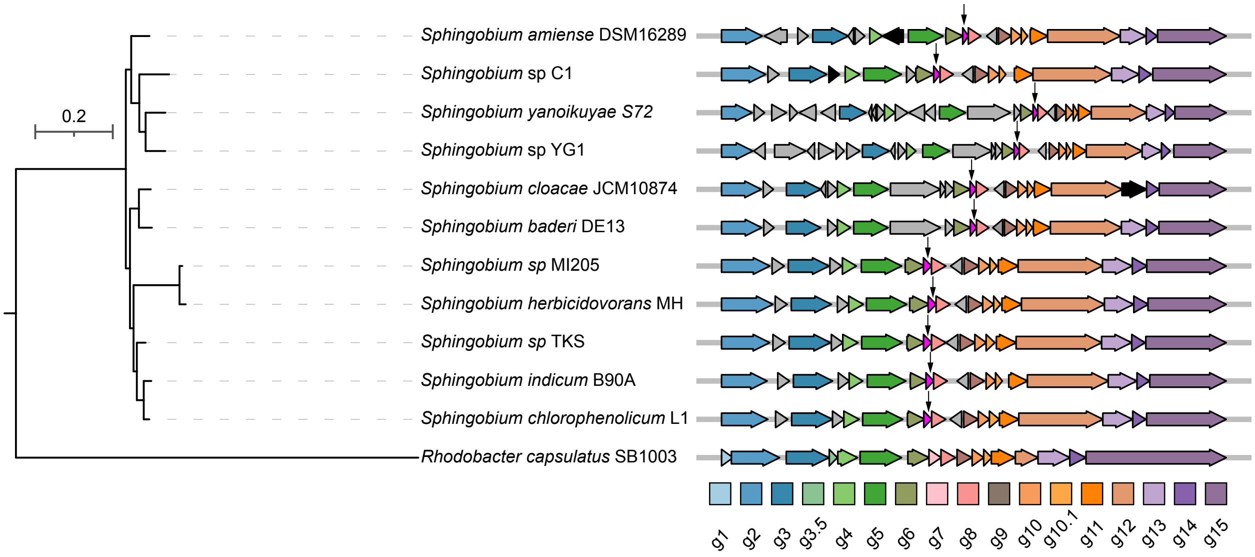

Supplemental Figure S5. Gene neighborhood of the RcGTA gene *g7* and of the putative *g7* replacement gene in 11 *Sphingomonadales* spp. Only region corresponding to the RcGTA 'head-tail' cluster is depicted, with each gene represented by an arrow scaled relative to its length within each cluster. The RcGTA genes (*g1*-*g15*) are color-coded and their homologs in *Sphingomonadales* are shown in the same color. Putative *g7* replacements in *Sphingomonadales* are shown in magenta and marked with an arrow. Pseudogenes are colored in black, while genes without an established relationship to GTA production are shown in gray. Phylogenetic tree is a subtree extracted from the reference phylogeny. The scale bar corresponds to the number of substitutions per site.
