# Supplementary material for: Selection for Translational Efficiency in Genes Associated with Alphaproteobacterial Gene Transfer Agents
Source: mSystems. 2022 Nov 14;7(6):e00892-22. doi: 10.1128/msystems.00892-22 (PMC9765227; doi:10.1128/msystems.00892-22)
Supplement: TABLE S3 [file msystems.00892-22-s0009.pdf]

Supplemental Table S3. Decisions behind choosing the RcGTA 'head-tail' cluster homologs for the reference GTA gene set. The GTA genes selected for the reference set are highlighted in orange. Functional annotations are based on the descriptions in the RefSeq database records, unless noted otherwise.

GTA gene	RcGTA RefSeq ID	RcGTA functional annotation	Included?	Reason for a gene exclusion
g1	WP_013067406.1	small terminase [1]		Homologs are detected only in *Rhodobacterales* order
g2	WP_031321187.1	terminase family protein	Yes	
g3	WP_013067408.1	phage portal protein	Yes	
g3.5	WP_031323538.1	hypothetical protein		The gene is < 300 nucleotides in length
g4	WP_013067410.1	HK97 family phage prohead protease	Yes	
g5	WP_037091462.1	phage major capsid protein	Yes	
g6	WP_013067412.1	adaptor protein [2]	Yes	
g7	WP_013067413.1	head-tail adaptor protein		Detected only in 10% of *Sphingomonadales* genomes
g8	WP_013067414.1	tail terminator protein [2]	Yes	
g9	WP_013067415.1	phage major tail protein, TP901-1 family		Patterns of the selection for translational efficiency are inconsistent with those of other GTA genes
g10	WP_013067416.1	gene transfer agent family protein	Yes	
g10.1	WP_013067417.1	phage tail assembly chaperone		The gene is < 300 nucleotides in length
g11	WP_013067418.1	phage tail tape measure protein	Yes	
g12	WP_013067419.1	distal tail protein [2]	Yes	
g13	WP_013067420.1	baseplate hub protein [2]	Yes	
g14	WP_013067421.1	peptidase	Yes	
g15	WP_013067422.1	glycoside hydrolase/phage tail family protein	Yes	

1. Sherlock, D., J.X. Leong, and P.C.M. Fogg, *Identification of the First Gene Transfer Agent (GTA) Small Terminase in Rhodobacter capsulatus and Its Role in GTA Production and Packaging of DNA.* J Virol, 2019. **93**: e01328-19.
2. Bardy, P., et al., *Structure and mechanism of DNA delivery of a gene transfer agent.* Nat Commun, 2020. **11**: 3034.
