# Supplementary material for: Selection for Translational Efficiency in Genes Associated with Alphaproteobacterial Gene Transfer Agents
Source: mSystems. 2022 Nov 14;7(6):e00892-22. doi: 10.1128/msystems.00892-22 (PMC9765227; doi:10.1128/msystems.00892-22)
Supplement: FIG S6 [file msystems.00892-22-s0006.pdf]

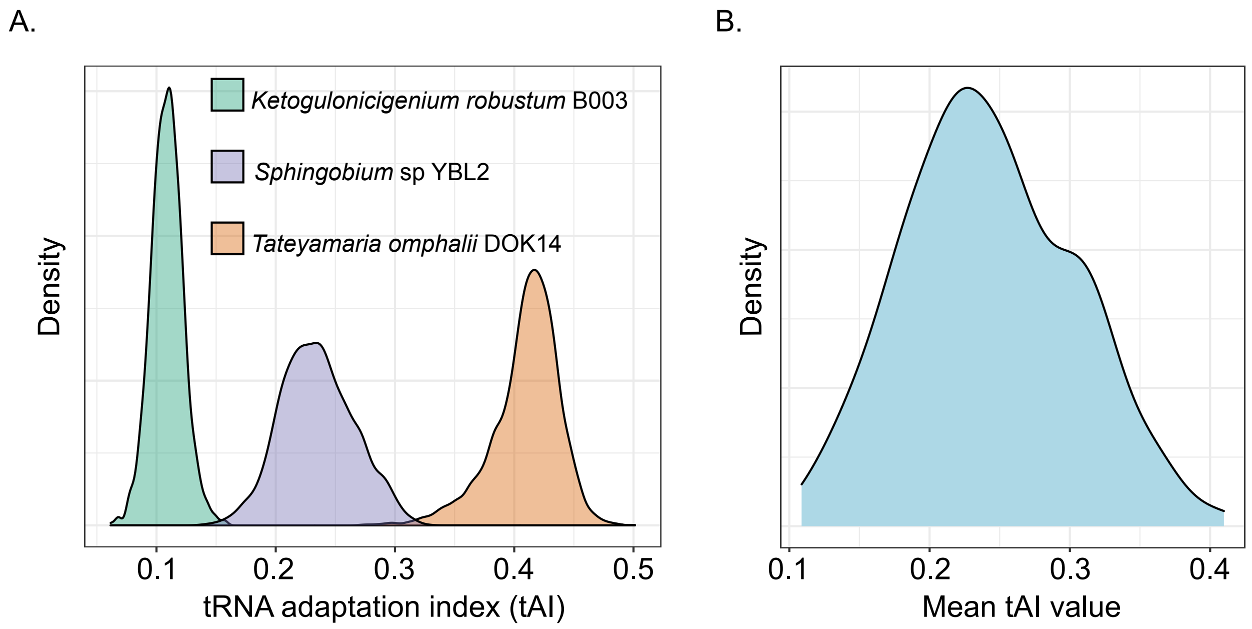

Supplemental Figure S6. Distribution of tAI values in protein-coding genes of the analyzed genomes. Only genes at least 300 nucleotides in length were included. **A.** Distribution of tAI values of genes in three representative alphaproteobacterial genomes, selected to have the lowest, the median, and the highest mean tAI value among 208 genomes. **B.** Distribution of the average genomic tAI values across 208 genomes.
