# Supplementary material for: Selection for Translational Efficiency in Genes Associated with Alphaproteobacterial Gene Transfer Agents
Source: mSystems. 2022 Nov 14;7(6):e00892-22. doi: 10.1128/msystems.00892-22 (PMC9765227; doi:10.1128/msystems.00892-22)
Supplement: TABLE S4 [file msystems.00892-22-s0010.pdf]

Supplemental Table S4. Significance and slope of the fit of the phylogenetic generalized least squares (PGLS) models between the reference GTA genes and the *g9* gene.

Reference GTA gene	p-value	Slope
g2	0.01287	0.18307
g3	0.00145	0.20857
g4	0.00099	0.25720
g5	2.23E-06	0.28841
g6	0.00511	0.23485
g8	0.00645	0.20851
g10	0.12627	0.08297
g11	0.00061	0.21435
g12	0.00031	0.31015
g13	0.00162	0.26218
g14	0.03469	0.14122
g15	0.00138	0.27938
